# In-Hospital Outcomes of Left Atrial Appendage Occlusion Among Cancer Patients with Atrial Fibrillation: A Nationwide U.S. Study

**DOI:** 10.3390/cancers17081331

**Published:** 2025-04-15

**Authors:** Gilad Margolis, Lev Vishnevskiy, Adam Folman, Mark Kazatsker, Ariel Roguin, Eran Leshem

**Affiliations:** 1 Division of Cardiovascular Medicine, Hillel Yaffe Medical Center, The Rappaport Faculty of Medicine, Technion—Israel Institute of Technology, Haifa 3109601, Israel; giladm@hymc.gov.il (G.M.); lev.vishnevskiy@gmail.com (L.V.); adamf@hymc.gov.il (A.F.); markka@hymc.gov.il (M.K.); arielr@hymc.gov.il (A.R.); 2 Cardiac Electrophysiology Unit, Hillel Yaffe Medical Center, Hadera 3810101, Israel

**Keywords:** atrial fibrillation, left atrial appendage occlusion, malignancy

## Abstract

**Background**: Atrial fibrillation (AF) is frequently observed in cancer patients, driven by mutual comorbidities and increasing the risk of thromboembolic events. Impediments can hinder the utilization of anticoagulants among patients with malignancy—drug interactions with chemotherapy, renal dysfunction, drug intolerance, and increased bleeding risk. Left atrial appendage occlusion (LAAO) is an effective and safe non-pharmacological approach to prevent thromboembolic complications when anticoagulants are not suitable. Cancer patients were generally excluded from the original LAAO trials, and current safety and efficacy in cancer patients remain uncertain. **Methods**: This retrospective study utilized the National Inpatient Sample (NIS) database to analyze in-hospital outcomes of LAAO in US patients with and without cancer between 2016 and 2019. Patient demographics, comorbidities, procedures, and in-hospital outcomes were extracted using ICD-10-CM codes. **Results**: Among 12,273 hospitalizations for LAAO across the US representing an estimated 61,365 LAAO procedures, 2.2% (1365 cases) were performed in cancer patients. Older age, male gender, chronic kidney disease, prior stroke, and anemia were more prevalent in the cancer group, with 785 (58%) having a solid malignancy and 580 (42%) having a hematologic malignancy. Compared to non-cancer patients, cancer patients exhibited a higher rate of in-hospital complications (8.8% vs. 5.7%; *p* < 0.001), primarily driven by acute kidney injury (4.4% vs. 2.4%; *p* = 0.002), acute heart failure (3.7% vs. 2.6%; *p* = 0.012), and cardiac tamponade (1.5% vs. 0.8%; *p* = 0.006). No significant differences were observed in vascular complications, periprocedural stroke, or in-hospital mortality. Average length of stay (LOS) was longer in cancer patients (1.4 ± 2.7 days vs. 1.8 ± 2.5 days; *p* < 0.001). **Conclusions**: This nationwide study found that cancer patients undergoing LAAO had an increased rate of in-hospital complications, particularly acute kidney injury, heart failure, and tamponade, but no increase in in-hospital mortality was observed. Further research is needed to evaluate the long-term safety and efficacy of LAAO for managing embolic prevention in this complex patient population.

## Simple Summary

Safety of Left Atrial Appendage Occlusion (LAAO) in patients with cancer is not defined. Using the National Inpatient Sample database, in-hospital outcomes of LAAO in patients with and without cancer were analyzed, revealing that LAAO is increasingly performed also in cancer patients, accompanying the general LAAO procedural rise in the USA, as an alternative to anticoagulants for stroke prevention in atrial fibrillation. These cancer patients, who are often older and have more comorbidities, exhibited higher rates of in-hospital complications, specifically acute kidney injury, heart failure, and cardiac tamponade, though no increase in mortality was observed. This association with increased risk highlights the need for further research to evaluate the long-term safety and efficacy of LAAO in this complex patient population.

## 1. Introduction

Atrial fibrillation (AF) is a growing clinical concern with a rising prevalence, often co-occurring with cancer due to shared risk factors, with up to one in four patients having both [1]. Moreover, cancer therapies with cardiovascular toxicity may increase the risk of AF [2]. This intersection of arrhythmia and malignancy presents a complex and significantly escalating challenge in managing thromboembolic and hemorrhagic risks [3], posing a dual threat to patients’ overall health. While anticoagulation is crucial for stroke prevention in AF, its use in cancer patients is often limited by drug interactions (including with chemotherapy and biological treatments) [4], INR control issues [5], renal dysfunction, and bleeding risks from both blood dyscrasia and the presence of metastases [6]. The thrombogenic nature of malignancy necessitates anticoagulant therapy in AF even with a low CHA2DS2-VASc score [7,8].

In light of these complexities, clinicians explore alternatives to traditional oral anticoagulation therapy for cancer patients with AF. Left atrial appendage occlusion (LAAO) offers a potential alternative for stroke prevention in individuals unsuitable for oral anticoagulation [9]. However, the safety and efficacy of LAAO in cancer patients remain uncertain, especially given their exclusion from pivotal LAAO trials [10,11]. While endovascular treatment is still mainly reserved for those unsuitable for oral anticoagulants, cancer patients are currently undergoing LAAO as an alternative to anticoagulant treatment despite minimal supporting data [12]. Recent studies using the U.S. Nationwide Readmissions Database (NRD) have yielded mixed results regarding LAAO outcomes in this population [13,14], with no evidence of increased readmission, but higher periprocedural complications and major adverse events. To address this question, the National Inpatient Sample (NIS) database was utilized to analyze in-hospital outcomes of LAAO in cancer patients within the U.S. We aimed to identify factors influencing procedural success, postoperative complications, and mortality, providing insights to guide clinical decision making regarding LAAO in this high-risk cohort of cancer patients.

## 2. Methods

Data from the National Inpatient Sample (NIS), a component of the Healthcare Cost and Utilization Project (HCUP) maintained by the Agency for Healthcare Research and Quality (AHRQ), were utilized in the current study. The NIS encompasses a 20% stratified sample of all inpatient discharges from U.S. hospitals, providing a national perspective on hospitalizations [15,16]. Only data regarding the initial hospitalization, including in-hospital data, complications, and mortality, are coded, and therefore, no follow up data and long-term outcomes can be assessed. NIS data were collected between 1 January 2016 and 31 December 2019. This study included patients aged 18 years or older who were diagnosed with AF, underwent LAAO, and were identified using specific ICD-10-CM codes. The cohort was divided according to the presence of malignancy, as coded for each patient.

Data collected from the NIS encompassed patient demographics (age, sex, and race/ethnicity), a CHA2DS2-VASc score, comorbidities, secondary diagnoses, procedures, length of stay, and in-hospital mortality (Appendix A, Table A1). The Deyo–Charlson Comorbidity Index (Deyo-CCI), a validated measure of comorbidity burden, was calculated using ICD-10-CM codes (Appendix A, Table A2) [17,18]. The ATRIA Bleeding Score is a clinical prediction rule for estimating the risk of bleeding in a patient about to commence anticoagulation, with a maximum score of 10 points and 5 variables including anemia, severe renal disease, age ≥75 years, prior bleeding, and hypertension. Scores above 5 indicate a high risk for bleeding [19]. Cancer diagnosis was established with relevant ICD-10-CM codes (Appendix A, Table A3).

The primary outcome of this study was in-hospital complications, with secondary outcomes including individual components of the primary outcome, adverse events during hospitalization, length of stay, and in-hospital mortality. The NIS database includes only de-identified data; therefore, studies on the NIS registry are exempt from institutional review by the local Human Research Committee. 

Statistical analyses were conducted using SPSS^®^ software version 23. Frequencies and proportions of demographic, clinical, and hospital-related variables were calculated and weighted to reflect national estimates using discharge sample weights provided by the NIS/HCUP. Comparisons between the cancer and non-cancer groups were performed using the appropriate statistical tests: Pearson’s chi-square test for categorical variables; for continuous variables, each was tested for normality of distribution using Q-Q plots and histograms. Normally distributed variables were reported as mean ± SD, and independent samples *t*-tests were applied. If not normally distributed, the variable was reported using the median and the IQR using the Mann–Whitney test. Generalized linear models were employed to analyze annual trends, utilizing a linear regression mode for overall LAAO procedural yearly trends, and a binary (binominal) logistic regression mode for the annual rates of cancer patients among LAAO recipients. A *p*-value less than 0.05 was considered statistically significant.

## 3. Results

**Figure 1:**
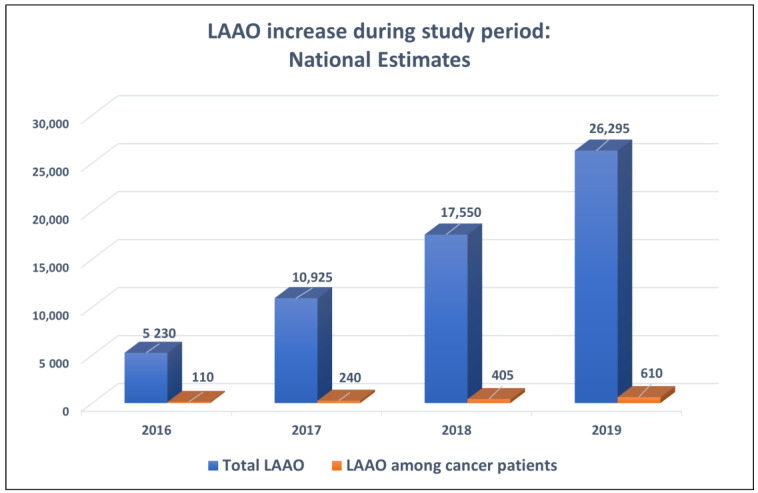
LAAO procedural growth in the U.S. during the study period according to the NIS database, and the accompanying LAAO procedures in cancer patients.

This study analyzed a total of 12,273 hospitalizations for LAAO (12,000 patients without cancer and 273 with cancer) across the United States between 2016 and 2019, representing an estimated 61,365 hospitalizations after applying weighting methods. A gradual increase in total LAAO procedural volume (*p* < 0.001) was observed within the NIS during the study period, and that general increase was accompanied by an increase in the number of procedures performed in cancer patients, although cancer patients were only 2.1–2.3% of the annual cohorts (*p* = 0.29) (Figure 1).

The cohort predominantly comprised males (61%), with a mean age of 77 years for non-cancer patients and 78 years for cancer patients. Of the estimated cohort, 1365 patients (2.2%) had a coded cancer diagnosis.

Table 1 presents the baseline characteristics of the study population, stratified by cancer status. Compared to non-cancer patients, those with cancer exhibited a higher prevalence of older age, male gender, chronic kidney disease, prior stroke, and anemia. While the CHA2DS2-VASc score differed statistically but not clinically between groups, cancer patients demonstrated higher ATRIA bleeding scores and had a higher CCI.

**Figure 2:**
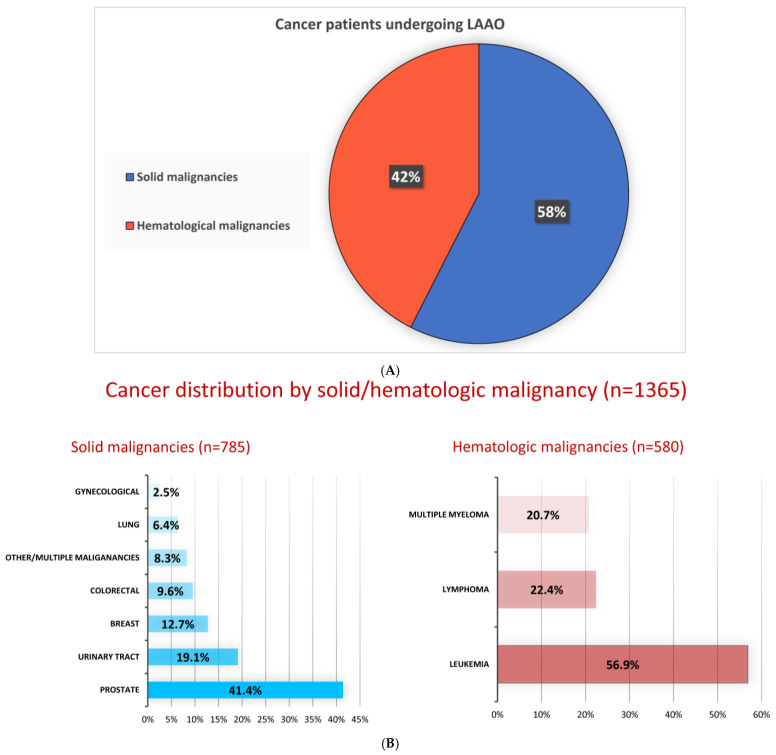
Distribution of solid versus hematological malignancies among cancer patients undergoing LAAO (**A**); specific percentiles of different malignancies among this cohort (**B**).

Among cancer patients undergoing LAAO, 58% had solid malignancies, with prostate and bladder cancer being the most frequent. Hematologic malignancies accounted for 42% of cases, with leukemia being the most common (Figure 2).

**Figure 3:**
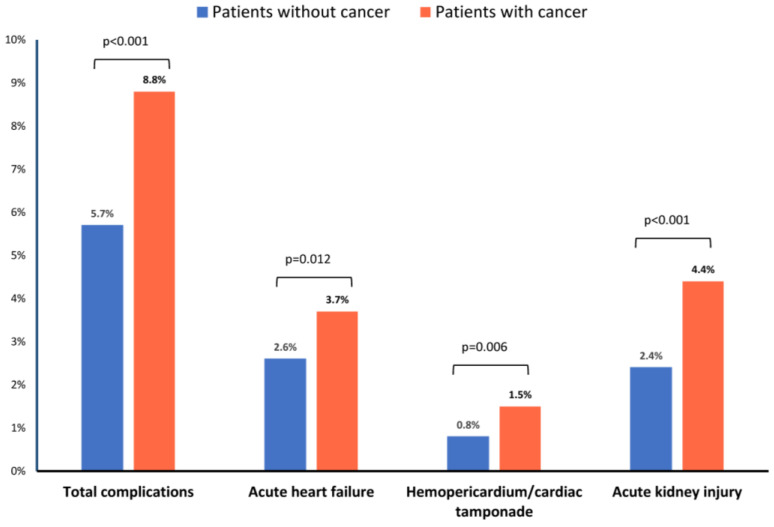
Increased rates of in-hospital complications among patients with cancer undergoing LAAO.

In-hospital complications occurred in 5.7% of all patients (Table 2). Notably, patients with cancer experienced a significantly higher rate of in-hospital complications (8.8%) compared to those without cancer (5.7%, *p* < 0.001). This increased complication rate in the cancer group was primarily attributed to a higher incidence of acute kidney injury (4.4% vs. 2.4%; *p* = 0.002), acute heart failure (3.7% vs. 2.6%; *p* = 0.012), and cardiac tamponade (1.5% vs. 0.8%; *p* = 0.006) (Figure 3).

While vascular and respiratory complications were observed, no significant differences in these rates or in periprocedural stroke or in-hospital mortality were found between the cancer and non-cancer groups. The average length of hospital stay was significantly longer for cancer patients (1.8 ± 2.5 days) compared to non-cancer patients (1.4 ± 2.7 days).

## 4. Discussion

This study aimed to investigate the in-hospital outcomes of LAAO in a large cohort of patients with atrial fibrillation (AF) utilizing the National Inpatient Sample (NIS) database and to compare these acute outcomes among patients with and without cancer.

Many cancer patients have different impediments to the use of oral anticoagulation, and the utilization of LAAO in this group occurs in around 2.2% of cases. While higher risks are observed in this population, the increased growth of LAAO procedures in general, especially in the U.S., will also likely increase among patients with concomitant malignancy. This is prone to occur despite their exclusion from the initial randomized controlled studies and the current incomplete data regarding safety and efficacy in this population. The current findings among the large NIS dataset provide additional crucial data regarding the real-life safety of the procedure in cancer patients. However, due to the higher thromboembolic risk in cancer patients, the long-term efficacy should also be studied but is beyond the scope of this manuscript and database.

The majority of baseline differences in this real-life registry of invasive procedures that have been performed are in line with the comorbidities associated with malignancy. However, the lower number of females in the group of cancer patients who underwent LAAO may represent selection bias of the implanting physicians. Lower utilization rates among females in invasive cardiac procedures are common in both randomized controlled trials and in prospective cohorts, and this may be emphasized among cancer patients due to the assumed increased vulnerability.

Our findings demonstrate a significantly higher rate of in-hospital complications in cancer patients undergoing LAAO compared to those without cancer. This increased risk was primarily driven by a higher incidence of acute kidney injury, heart failure, and tamponade. While the overall rates of vascular complications, periprocedural stroke, and in-hospital mortality were low in both groups, the longer length of stay observed in cancer patients highlights the potential for increased resource utilization and healthcare costs.

These findings align with previous studies suggesting a higher risk of adverse events in cancer patients undergoing LAAO [13,14]. Yet, these prior studies showed conflicting results regarding specific complications that are increased in this vulnerable population. While both of the previous studies were based on the National Readmissions Database (NRD), one found a myriad of in-hospital complications that were associated with LAAO in cancer, while the other revealed an increase solely in stroke/TIA, but not the other outcomes that were assessed. Our study extends these observations by providing a larger cohort with a comprehensive analysis of in-hospital complications in a different large, U.S. nationwide representative cohort.

A recent long-term, observational, single-center study spanning close to 14 years showed no significant differences in periprocedural complications [20]. However, despite the relatively large number of patients with cancer in this study, patients with cancer comprised a quarter of the cohort (93/361), reflecting different LAAO utilization patterns compared with those in the current real-world observation (2.2%). This may be the result of local practices that include cancer patients initially in the LAAO program, but do not reflect the current standard of care.

The identification of acute kidney injury, heart failure, and tamponade complications as the primary drivers of increased risk in cancer patients has important clinical implications. These complications may be related to the underlying malignancy, its treatment, or the immunosuppressive effects of cancer. Multifactorial causes can contribute to kidney injury in the cancer population, including treatment impact and the moderate deterioration of kidney function from the malignancy combined with the need to inject contrast agents during the LAAO procedure. Likewise, cardiovascular toxicity from anticancer treatments may contribute to the development of heart failure, especially in the setting of intracardiac procedures usually requiring anesthesia. In addition, they emphasize the susceptibility of the cancer population to procedural mechanical complications such as cardiac tamponade, especially those with hematological malignancy, due to the combination of associated anemia and blood dyscrasia with the delivery of heparin during the procedure to prevent systemic thromboembolism.

The observed differences in baseline characteristics between the cancer and non-cancer patients, such as higher rates of older age, male gender, chronic kidney disease, prior stroke, and anemia in the cancer group, may partially contribute to the increased risk of complications. These factors are known to be associated with increased morbidity and mortality in various clinical settings. The higher ATRIA bleeding score observed in cancer patients highlights the increased bleeding risk, despite no evidence of increased hemorrhage or vascular complications in cancer patients.

These results suggest the need for careful patient selection and risk stratification prior to LAAO when undertaking this invasive procedure in patients with malignancy. Assessments of bleeding risk, cancer treatment history, and concomitant medications are vital in this vulnerable population.

## 5. Limitations

Several limitations inherent to the NIS database should be acknowledged. As a retrospective administrative database containing discharge-level records, the NIS is susceptible to coding errors and inaccuracies. Notably, the lack of granular information regarding the exact cancer status within the NIS presents a significant limitation. This precludes the precise differentiation between patients with active versus inactive prior malignancy, although the actual coding of the malignancy diagnosis for an acute hospitalization is most probably reflective of the ongoing nature of the disease.

Furthermore, the study sample included a relatively small number of cancer patients undergoing LAAO procedures. The analysis was restricted to in-hospital events, limiting the ability to assess long-term outcomes such as stroke, bleeding, and all-cause mortality. The absence of detailed clinical information, such as specific cancer types, cancer stage, medication use (including the antineoplastic regimen), and blood test results, within the NIS database further restricts the ability to draw definitive conclusions about the impact of malignancy on LAAO outcomes.

Despite these limitations, this study offers valuable insights into the real-world experiences of cancer patients who underwent LAAO by leveraging a large, nationally representative dataset. These findings, however, should be interpreted cautiously and require further validation through prospective studies with a more comprehensive data collection procedure.

## 6. Conclusions

This study provides a signal that cancer patients undergoing LAAO may experience higher rates of in-hospital complications, particularly acute kidney injury and cardiac complications such as tamponade and acute heart failure, compared to non-cancer patients. These findings suggest careful patient selection and close monitoring for complications in this vulnerable population. Further research, including prospective studies with longer follow-up periods, is warranted to further elucidate the long-term risks and benefits of LAAO in cancer patients with AF.

## Figures and Tables

**Table 1:** Baseline characteristics of patients with and without cancer undergoing left atrial appendage occlusion (LAAO).

Variable	Patients Without Cancer (N = 60,000)	Patients with Cancer (NE = 1365)	*p* Value
Age (years) (median [IQR])	77 (71–82)	78 (74–82)	<0.001
Female	25,220 (42%)	395 (29%)	<0.001
White ethnicity	50,805 (87%)	1150 (87%)	0.915
Diabetes mellitus	20,240 (34%)	420 (31%)	0.022
Hypertension	27,520 (46%)	605 (44%)	0.258
Heart failure	23,535 (39%)	525 (38%)	0.568
Valvular heart disease	10,155 (17%)	255 (19%)	0.087
Chronic kidney disease	14,380 (24%)	365 (27%)	0.018
Peripheral artery disease	5250 (9%)	120 (9%)	0.958
Prior stroke	2125 (3.5%)	65 (5%)	0.016
Prior myocardial infarction	7895 (13%)	155 (11%)	0.051
Anemia	8070 (14%)	270 (20%)	<0.001
Obesity (BMI > 30)	7135 (12%)	185 (14%)	0.061
Chronic pulmonary disease	10,805 (18%)	225 (16%)	0.147
Dementia	1795 (3%)	50 (4%)	0.151
Hypertrophic cardiomyopathy	400 (1%)	<10	NA
CHA_2_DS_2_ VASc score (mean ± SD)	3.3 ± 1.1	3.2 ± 1.1	0.035
ATRIA bleeding score (mean ± SD)	2.2 ± 1.5	2.6 ± 1.6	<0.001
Charlson comorbidity index (median [IQR])	1 (0–3)	3 (2–5)	<0.001
Income percentile			
0–25	12,835 (22%)	205 (15%)	<0.001
26–50	15,340 (26%)	325 (24%)	
51–75	16,500 (28%)	400 (30%)	
76–100	14,475 (24%)	425 (31%)	
Teaching hospital	53,000 (88%)	1200 (88%)	0.632
Hospital region			0.001
South/west	23,315 (39%)	590 (43%)	
Midwest/northeast	36,685 (61%)	775 (57%)	

NE—national estimate. NA—non applicable.

**Table 2:** Periprocedural outcomes of left atrial appendage occlusion (LAAO) in patients with and without cancer.

Variable	Patients Without Cancer (NE = 60,000)	Patients with Cancer (NE = 1365)	*p* Value
**Total complications**	3400 (5.7%)	120 (8.8%)	<0.001
**Cardiac complications**			
Cardiogenic shock	150 (0.3%)	None	NA
Cardiac arrest	50 (0.1%)	None	NA
Acute heart failure	1545 (2.6%)	50 (3.7%)	0.012
Hemopericardium/cardiac tamponade	475 (0.8%)	20 (1.5%)	0.006
**Vascular complications**			
Bleeding requiring blood transfusion	70 (0.1%)	≤10	NA
Vascular injury	235 (0.4%)	≤10	NA
**Sepsis**	115 (0.2%)	None	NA
**Respiratory complications**			
Post-procedure respiratory failure	140 (0.2%)	≤10	NA
Diaphragmatic disorder	≤10	None	NA
Invasive ventilation >24 h	40 (0.1%)	None	NA
**Neurologic complications (stroke)**	≤10	None	NA
**Acute kidney injury**	1445 (2.4%)	60 (4.4%)	<0.001
**Length of stay** (mean ± SD)	1.4 ± 2.7	1.8 ± 2.5	<0.001
**In-hospital mortality**	95 (0.2%)	None	NA

NE—national estimate. NA—non applicable.

## Data Availability

The data presented in this study are available from the AHRQ agency at their public domain (https://hcup-us.ahrq.gov/db/nation/nis/nisdbdocumentation.jsp; accessed 7 April 2025). The raw data supporting the conclusions of this article will be made available by the authors on request.

## Appendix A

**Table A1:** ICD-10 CM/PCS codes used in data extraction.

ICD-10 CM/PCS Codes	Condition/Procedure
**Codes for inclusion criteria.**	
I480, I481, I482, I4891	Atrial fibrillation primary diagnosis
02L73DK	Left atrial appendage occlusion (LAAO)
**Codes for baseline characteristics and comorbidities not included in Deyo-CCI.**	
I10	Hypertension
G4730, G4733	Obstructive sleep apnea
E66.01, E66.2, E66.09, E66.1, E66.8, E66.9, Z68.4, Z68.3	Obesity
D50.x, D51.x, D52.x, D53.x, D64.x	Anemia
I42.1, I42.2	Hypertrophic cardiomyopathy
I351, I350, I352, I358, I359, I340, I341, I342, I348, I349, I360, I361, I362, I368, I369, I050, I051, I052, I058, I059, I060, I061, I062, I068, I069, I070, I071, I072, I078, I079,I080, I081, I082, I083, I088, I089	Valvular heart disease
I60.x, I61-2.x, H356, H313, H431, H0523, I8501, I8511, K2901, K2921, K2931, K2941, K2951, K2961, K2971, K2981, K2991, K920, K921, K922, K5701, K5721, K5731, K5733, K5741, K5781, K625, M250, M7981, K661	Prior hemorrhage diagnosis
**Procedural complication-related ICD codes—vascular complications.**	
K916, K9184, I974, G9752, I97618, I97618, N99821 30233H, 30233N	Hemorrhage/hematoma/blood transfusion
T81719A. T81718A, T81711A, T81710A, I975, I976, S750, S751, S651, I770, I724.	Vascular injury
**Procedural complication-related ICD codes—cardiac complications.**	
T8111XA, T8110XA	Shock
I9712x, I977x	Cardiac arrest
I9711x, I9713x, I9719x	Acute heart failure
I312	Hemopericardium
I314	Cardiac temponade
**Procedural complication-related ICD codes—respiratory complications.**	
J9582, J9588, J9589 5A1935Z, 5A1945Z, 5A1955Z, 0B110F4, 0B113F4	Post-procedure respiratory failure or intubation > 24 h
J986	Diaphragmatic disorder
**Procedural complication-related ICD codes—neurologic complications.**	
I9781x, I9782x	Stroke
**Procedural complication-related ICD codes—infectious complications.**	
T80211A, T8149XA, T8144XA, T8112XA, A40x, A41x, R6520, R6521	Bacteremia and sepsis

**Table A2:** ICD-10 CM/PCS codes for conditions incorporated in Deyo-CCI, and scoring system used to compute CCI scores.

ICD-10 CM Codes	Condition	Score
I21.x, I22.x, I25.2	Myocardial infarction	1
I11.0, I13.0, I13.2, I25.5, I42.0, I42.5-I42.9, I43.x, I50.x, P29.0	Heart failure	1
I70.x, I71.x, I73.1, I73.8, I73.9, I77.1, I79.0, I79.1, I79.8, K55.1, K55.8, K55.9, Z95.8, Z95.9	Peripheral vascular disease	1
G45.x, G46.x, H34.0x, H34.1x, H34.2x, I60.x-I68.x	Cerebrovascular disease	1
F01.x-F03.x, F04, F05, F06.1, F06.8, G13.2, G13.8, G30.x, G31.0x, G31.1, G31.2, G91.4, G94, R41.81, R54	Dementia	1
J40.x-J47.x, J60.x-J67.x, J68.4, J70.1, J70.3	Chronic pulmonary disease	1
M05.x, M06.x, M31.5, M32.x-M34.x, M35.1, M35.3, M36.0	Rheumatologic disease	1
K25.x-K28.x	Peptic ulcer disease	1
B18.x, K70.0-K70.3, K70.9, K71.3-K71.5, K71.7, K73.x, K74.x, K76.0, K76.2-K76.4, K76.8, K76.9, Z94.4	Mild liver disease	1
Main codes: E08, E09, E10, E11, E13. Relevant subcodes: E**.0x, E**.1x, E**.6x, E**.8x, E**.9x	Diabetes without chronic complications	1
Main codes: E08, E09, E10, E11, E13. Relevant subcodes: E**.2x, E**.3x, E**.4x, E**.5x	Diabetes with chronic complications	2
G04.1, G11.4, G80.0, G80.1, G80.2, G81.x, G82.x, G83.x	Hemiplegia or paraplegia	2
C0x.x, C1x.x, C2x.x, C30.x-C34.x, C37.x-C41.x, C43.x, C45.x-C58.x, C60.x-C63.x,C76.x, C80.1, C81.x-C85.x, C88.x, C9x.x	Any malignancy including leukemia and lymphoma except malignant nonmelanoma of the skin	2
I85.0x, I86.4, K70.4x, K71.1x, K72.1x, K72.9x, K76.5, K76.6, K76.7	Moderate or severe liver disease	3
I12.0, I13.11, I13.2, N18.5, N18.6, N19.x, N25.0, Z49.x, Z99.2	Renal disease, severe	3
B20.x	HIV infection	3
C77.x-C79.x, C80.0, C80.2	Metastatic solid tumor	6
B37.x, C53.x, B38.x, B45.x, A07.2, B25.x, G93.4x, B00, B39.x, A07.3, C46.x, C81-96, A31.x, A15-19, B59, Z87.01, A81.2, A02.1, B58.x, R64	Acquired immunodeficiency syndrome (AIDS)	6

**Table A3:** ICD-10 CM and ICD-9 CM codes for primary cancer site diagnosis.

ICD-10 CM Codes	Cancer Site
C00–C14, C30–C33	Head and neck
C15	Esophagus
C16	Stomach
C18-C20	Colorectal
C17, C21, C24, C26	Other GI
C22	Liver
C23	Biliary
C25	Pancreatic
C34	Lung
C37–C39	Mediastinum
C40, C41, C45–C49	Connective tissue
C42	Melanoma
C50	Breast
C51–C58	Gynecological
C61	Prostate
C60, C62, C63	Male genital
C64–C68	Urinary tract
C69–C72	Central nervous system
C73	Thyroid
C74–C75	Other endocrine
C81	Hodgkin lymphoma
C82–C86, C88, C89	Non-Hodgkin lymphoma
C90	Multiple myeloma
C91–C96	Leukemia
C7A	Neuroendocrine

## References

[1] Melloni C., Shrader P., Carver J., Piccini J.P., Thomas L., Fonarow G.C., Ansell J., Gersh B., Go A.S., Hylek E. (2017). Management and outcomes of patients with atrial fibrillation and a history of cancer: The ORBIT-AF registry. Eur. Hear. J. Qual. Care Clin. Outcomes.

[2] Fradley M.G., Beckie T.M., Brown S.A., Cheng R.K., Dent S.F., Nohria A., Patton K.K., Singh J.P., Olshansky B., Arteriosclerosis T.C.O. (2021). Recognition, prevention, and management of arrhythmias and autonomic disorders in cardio-oncology: A scientific statement from the American heart association. Circulation.

[3] Poénou G., Tolédano E., Helfer H., Plaisance L., Happe F., Versini E., Diab N., Djennaoui S., Mahé I. (2023). Assessment of bleeding risk in cancer patients treated with anticoagulants for venous thromboembolic events. Front. Cardiovasc. Med..

[4] Mosarla R.C., Vaduganathan M., Qamar A., Moslehi J., Piazza G., Giugliano R.P. (2019). Anticoagulation Strategies in Patients With Cancer. JACC Rev. Cardiovasc. Interv..

[5] Pokorney S., Simon D.N., Thomas L., Fonarow G., Kowey P., Chang P., Singer D., Ansell J., Blanco R., Gersh B. (2015). The Myth of the Stable INR Patient: Results from ORBIT-AF. J. Am. Coll. Cardiol..

[6] Lin R.J., Green D.L., Shah G.L. (2018). Therapeutic Anticoagulation in Patients with Primary Brain Tumors or Secondary Brain Metastasis. Oncologist.

[7] Chu G., Seelig J., Cannegieter S.C., Gelderblom H., Hovens M.M., Huisman M.V., van der Hulle T., Trines S.A., Vlot A.J., Versteeg H.H. (2023). Atrial fibrillation in cancer: Thromboembolism and bleeding in daily practice. Res. Pract. Thromb. Haemost..

[8] Margolis G., Goldhaber O., Kazatsker M., Kobo O., Roguin A., Leshem E. (2024). Atrial Fibrillation Catheter Ablation among Cancer Patients: Utilization Trends and In-Hospital Outcomes. J. Clin. Med..

[9] Naksuk N., Padmanabhan D., Yogeswaran V., Asirvatham S.J. (2016). Left Atrial Appendage: Embryology, Anatomy, Physiology, Arrhythmia and Therapeutic Intervention. JACC Clin. Electrophysiol..

[10] Holmes D.R., Reddy V.Y., Turi Z.G., Doshi S.K., Sievert H., Buchbinder M., Mullin C.M., Sick P. (2009). PROTECT AF Investigators. Percutaneous closure of the left atrial appendage versus warfarin therapy for prevention of stroke in patients with atrial fibrillation: A randomised non-inferiority trial. Lancet.

[11] Holmes D.R., Kar S., Price M.J., Whisenant B., Sievert H., Doshi S.K., Huber K., Reddy V.Y. (2014). Prospective randomized evaluation of the Watchman Left Atrial Appendage closure device in patients with atrial fibrillation versus long-term warfarin therapy: The PREVAIL Trial. J. Am. Coll Cardiol..

[12] Shabtaie S.A., Tan N.Y., Ward R.C., Lewis B.R., Yang E.H., Holmes D.R., Herrmann J. (2023). Left atrial appendage occlusion in patients with atrial fibrillation and cancer. J. Am. Coll. Cardiol. CardioOnc..

[13] Agarwal S., Guha A., Munir M.B., DeSimone C.V., Deshmukh A., Asad Z.U.A. (2023). Outcomes of patients with cancer undergoing percutaneous left atrial appendage occlusion. J. Interv. Card. Electrophysiol..

[14] Isogai T., Saad A.M., Abushouk A.I., Shekhar S., Kuroda S., Gad M.M., Wazni O.M., Krishnaswamy A., Kapadia S.R. (2020). Procedural and short-term outcomes of percutaneous left atrial appendage closure in patients with cancer. Am. J. Cardiol..

[15] HCUP National Inpatient Sample (NIS) Agency for Healthcare Research and Quality. https://www.hcup-us.ahrq.gov/nisoverview.jsp.

[16] Steiner C., Elixhauser A., Schnaier J. (2002). The healthcare cost and utilization project: An overview. Eff. Clin. Pract..

[17] Deyo R.A., Cherkin D.C., Ciol M.A. (1992). Adapting a clinical comorbidity index for use with ICD-9-CM administrative databases. J. Clin. Epidemiol..

[18] Radovanovic D., Seifert B., Urban P., Eberli F.R., Rickli H., Bertel O., A Puhan M., Erne P., on behalf of the AMIS Plus Investigators Validity of Charlson Comorbidity Index in patients hospitalised with acute coronary syndrome (2014). Insights from the nationwide AMIS Plus registry 2002–2012. Heart.

[19] Fang M.C., Go A.S., Chang Y., Borowsky L.H., Pomernacki N.K., Udaltsova N., Singer D.E. (2011). A new risk scheme to predict warfarin-associated hemorrhage: The ATRIA (Anticoagulation and Risk Factors in Atrial Fibrillation) Study. J. Am. Coll Cardiol..

[20] Tinoco M., Echarte-Morales J., Guerreiro C.E., Gil E.M.Á., Caneiro-Queija B., Barreiro-Pérez M., González-Ferreiro R., Fernández S., Ortiz-Saez A., Jiménez-Díaz V.A. (2024). Short- and long-term outcomes of percutaneous left atrial appendage occlusion in cancer patients. Int. J. Cardiol. Heart Vasc..

